# Aquaculture potential of Atlantic wolffish (*Anarhichas lupus*): stress and physiological responses to acute handling

**DOI:** 10.1007/s10695-025-01456-4

**Published:** 2025-02-01

**Authors:** Ida Hedén, Jonathan Armand Charles Roques, Marica Andersson, Niklas Warwas, Raneesha de Fonseka, Darragh Doyle, James Hinchcliffe, Elisabeth Jönsson, Kristina Sundell, Henrik Sundh

**Affiliations:** 1 Department of Biological and Environmental Sciences, University of Gothenburg, Box 463, 405 30 Gothenburg, Sweden; 2 Swedish Mariculture Research Center (SWEMARC), University of Gothenburg, Box 463, 405 30 Gothenburg, Sweden; 3 Blue Food Center, University of Gothenburg, Box 463, 405 30 Gothenburg, Sweden

**Keywords:** Atlantic wolffish, Stress, Cortisol, Diversification, Aquaculture

## Abstract

The Atlantic wolffish (*Anarhichas lupus*) is a cold-water species with the potential to diversify aquaculture in Northern countries. Few studies have investigated the stress physiology of Atlantic wolffish, and the current knowledge on stress in wolffish species is largely derived from the closely related spotted wolffish (*Anarhichas minor*). In the current study, Atlantic wolffish were exposed to handling stress mimicking common husbandry conditions in aquaculture such as repeated air exposure and net-chasing. Samples were taken prior to stress exposure (pre-stress; control) as well as 5- and 24-h post-stress. A series of primary and secondary acute stress response parameters were assessed: plasma cortisol, glucose and lactate levels, hematological indices (hemoglobin, Hb; hematocrit, Hct; mean corpuscular hemoglobin concentration, MCHC), and osmoregulatory capacity through plasma osmolality and gill Na^+^/K^+^ATPase (NKA) activity. Other secondary stress responses with implications for fish health and welfare are intestinal integrity and transport functions. These parameters were assessed using the Ussing chamber technique. The cortisol peak values were low in comparison to other fish species studied after acute handling stress and occurred as late as 24 h post-stress, suggesting that Atlantic wolffish is a slow and low cortisol responder. Plasma glucose remained stable, whereas lactate concentrations significantly decreased between 5 and 24 h after stress. There was no effect on pH, Hb, or Hct, although a significant increase in MCHC was found after 5 h and 24 h, originating from a small increase in Hb. This result suggests a minor increase in Hb synthesis after stress exposure. The intestinal integrity and transport functions as well as gill NKA-activity remained unchanged after stress exposure. In conclusion, Atlantic wolffish appears to exhibit a relatively moderate stress response, characterized by a slow and low primary stress response and minimal secondary effects following husbandry-related acute stress. These findings contribute to the understanding of the species’ potential for development as a candidate for marine, cold-water aquaculture.

## Introduction

Atlantic wolffish (*Anarhichas lupus*) and spotted wolffish (*Anarhichas minor*) have been proposed as promising species for aquaculture because of their large and well-developed eggs, the ability of the larvae to quickly accept formulated feed, their good temperament, and high-quality meat with good market potential (Albertsson et al. 2012; Foss et al. 2004; Moksness and Pavlov 1996; Pavlov and Moksness 1994). While the spotted wolffish has been shown to have higher growth rates and is already being commercially cultured (Le François et al. 2021a, b; Moksness 1994), Atlantic wolffish is the most interesting species for the diversification of Swedish aquaculture, since its native to Swedish waters (Albertsson et al. 2012; Ungfors 2015). Both the Atlantic and the spotted wolffish have shown great resilience in aquaculture environments. They grow efficiently in various tank systems such as shallow raceways, circular tanks, and flat bottom cages (Imsland et al. 2007; Mortensen et al. 2007; Øiestad 1999). They can be kept in as high densities as up to 90 kg/m^2^, although the optimal density has been suggested to be 25–30 kg/m^2^ in flow-through systems (Imsland et al. 2009; Jonassen 2002; Le François et al. 2013). Optimal temperature has been found to differ across life stages, with juveniles preferring higher temperatures than adults, and Atlantic wolffish was found to be more resilient to variation in temperature than spotted wolffish (Árnason et al. 2019, Hansen and Falk-Petersen 2002, Hinchcliffe et al. 2025a, Imsland et al. 2006).

In aquaculture, fish are occasionally exposed to procedures or environments that can be perceived as stressful, such as fluctuations in water quality, handling, air exposure, and aggressive behavior from conspecifics (Barton and Iwama 1991). In fish, as part of the primary stress response, a perceived stressor will activate the hypothalamic pituitary inter-renal axis (HPI axis). Corticotropin-releasing hormone (CRH) will be released from the hypothalamus and stimulate the release of adrenocorticotropic hormone (ACTH) from the pituitary gland. ACTH will in turn stimulate the production and release of cortisol from interrenal cells into the bloodstream (Barton 2002). Cortisol induces secondary stress responses in target tissues to help the fish cope with a short-term stressor by reallocating energy toward the different tissues targeted (such as muscles, gills, liver, intestine) to handle the stressor (Wendelaar Bonga 1997). This can result in a series of structural and functional changes in metabolism (including glucose and lactate), hematology, and hydromineral balance to face the stressor (Barton and Iwama 1991). In addition, the intestinal integrity, reflecting its barrier function, is another important physiological secondary stress indicator to both acute and chronic stress in other important aquaculture species (Knudsen et al. 2008; Olsen et al. 2005, 2008; Sundh et al. 2018, 2010, 2009; Vidakovic et al. 2016). Severe and/or chronic stress can further lead to tertiary effects such as impaired growth, reproduction, and disease resistance, for example through immuno-suppression and reduced epithelial barrier function, thus leading to poor health and welfare of the fish (Barton 2002; Barton and Iwama 1991). To prevent excessive cortisol levels, a negative feedback loop, initiated by the hormone, inhibits further CRH and ACTH release.

Few studies have been conducted to evaluate the stress responses in Atlantic wolffish, and to the authors’ knowledge, none has assessed the acute stress responses to common aquaculture handling procedures (Árnason et al. 2019; Foss et al. 2004; Hinchcliffe et al. 2025b). Studies on spotted wolffish suggest that they are stress-tolerant with a relatively low cortisol response in comparison to other farmed species, such as salmonids (Barton 2002; Lays et al. 2009; Le François et al. 2013). A previous study on spotted wolffish by Le François et al. (2013) found that the plasma cortisol concentration increased to approximately 30–35 ng/mL during handling and air exposure whereas crowding did not result in any cortisol response at all. Atlantic wolffish exposed to an acute or chronic increase in temperature to 15 °C did not show any sign of stress response connected to the HPI axis (Árnason et al. 2019; Hinchcliffe et al. 2025a). However, prolonged exposure to this high temperature reduced the intestinal barrier function thus indicating a secondary stress response (Hinchcliffe et al. 2025a).

The aim of the current study was to increase the understanding of the stress response in Atlantic wolffish and discuss that in relation to its potential as a commercially viable aquaculture species. The objectives were to clarify the impact of acute handling and air exposure on (1) the primary stress response, specifically cortisol release, and (2) the secondary stress responses including metabolites levels, hematological indices, osmoregulatory capacity, intestinal integrity, and transport functions.

## Material and methods

Wild fertilized Atlantic wolffish eggs caught during a bottom trawl survey by the Icelandic Marine and Freshwater Research Institute (Hafnarfjörður, Iceland) were subsequently brought to the Aquaculture Research Station of Grindavík, Iceland in early 2017, where they were hatched and kept up to the size of approximately 7 g. They were then transported to the animal facilities at the University of Gothenburg, Sweden. The fish were kept in a recirculating aquaculture system (RAS) with artificial seawater (salinity 30–33 ppt, sea salt Grotech, Ahorn, Germany), at 10 °C, and simulated 12:12-h light–dark photoperiod. The fish were fed at least 3 times per week until visual satiation with different size grades, according to the fish size, of commercial feed (Skretting Amber Neptune, Skretting, Stavanger, Norway). At the time of the experiment (April 2023), the fish were 6-year-old adults and had an average (± standard error) weight of 682 ± 44 g and a total length of 47 ± 0.75 cm. The fish were fasted for 3 days before stress exposure. The experiment started with measurements of pre-stress (control) stress levels where 10 fish were undisturbed for at least 48 h until netted and immediately euthanized by a lethal dose of Finquel (MS-222; 200 mg/L; Argent Chemical Laboratories Inc, Redmond, USA) and a sharp blow to the head. Thereafter, 10 fish were netted and subjected to air exposure (2 × 1 min) and subsequently transferred into a new holding tank, where they were exposed to 2 min of chasing. Following this procedure, 10 individuals were euthanized after 5 h and 10 other individuals were euthanized 24 h after this stress exposure. Weight and length were measured on all fish and blood samples were immediately taken by caudal puncture using a heparinized syringe. The hematocrit (Hct) and hemoglobin (Hb) concentrations were instantly measured, and the remaining blood was centrifuged at 4 °C for 5 min, at 10.000 × g (Thermo-scientific heraeus pico 17, Thermo Fisher Scientific, Waltham, USA), and the plasma was stored at − 80 °C until further analyses. A 1-cm cut was taken from the gill arch closest to the body (left side), put into an ice-cold SEI buffer, and stored at − 80 °C for NKA-activity measurements. The intestines of non-stressed controls and 24 h post-stress fish were sampled for Ussing chamber analysis. These fish were opened laterally, and the intestine was carefully cleaned from mesenteric fat using a tweezer. The intestine was divided into proximal and distal intestinal sections, as described in Hedén (2023), and opened using a blunt scissor and rinsed and stored in ice-cold Ringer’s solution modified for wolffish (160 mM NaCl; 2.0 mM KCl; 1.6 mM CaCl_2_ × 2H_2_O; 1 mM MgCl_2_ × 6H_2_O; 7 mM NaHCO_3_; 0.7 mM NaH_2_PO_4_ × 2H_2_O; 5 mM HEPES; 0,5 mM methionine; 10 mM D-glucose; 20 mM L-glutamine; pH 7.8) before mounted into the Ussing chambers.

### Plasma cortisol concentration

Plasma cortisol concentrations were measured in un-extracted plasma using a radioimmunoassay (RIA) described by Young (1986) and with the cortisol antibody validated by Sundh et al. (2011). In short, sheep cortisol antibody (Guildhay, Ltd. Guildford, Surrey, UK, no longer in activity) was used. Hydrocortisone [1,2,6,7-^3^H (N)] (NET 396, NEN Life Science Products, Boston, USA) was used as a tracer and the standard was prepared using hydrocortisone (Sigma-Aldrich, St. Louis, USA). Radioactivity was measured using a β-counter (Wallac 1409 Liquid Scintillation Counter, Turku, Finland). Samples below the detection limit (*n* = 2, both from pre-stress group) were assigned the value of the detection limit (0.5 ng/mL).

### Blood analysis

Hct was measured in duplicates by drawing blood into 80 μL heparinized capillaries that were sealed using critoseal clay. The capillaries were centrifuged for 5 min at 9.500 × g in a microcentrifuge (Haematokrit 210, Hettich, Tuttlingen, Germany). Hct was calculated as a percentage of total blood volume using a Hawksley reader (Hawksley & Sons Ltd., Lancing, UK). Hb was determined using a portable analyzer (Hb 201 + , Hemocue AB, Ängelholm, Sweden), and values were corrected for fish blood according to Clark et al. (2008). The mean corpuscular hemoglobin concentration (MCHC) was calculated using the equation MCHC = Hb × 100 / Hct.

### Plasma glucose, lactate, and osmolality

The concentrations of glucose and lactate were measured, in duplicate, using commercially available enzymatic kits (glucose hexokinase GHK20-kit, Sigma-Aldrich, St Louis, USA and lactate reagent set, enzymatic method, #2864 Instruchemie, Delfzijl, The Netherlands), according to a protocol adapted to a 96-well microplate reader (Schram et al. 2014). In short, for glucose, 10 µL of plasma was mixed with 200 µL reaction mixture, and the concentration was measured after 15 min incubation at room temperature in a microplate reader (SpectraMax 190, Molecular Devices Corp., Menlo Park, USA) at 340 nm. For lactate, 10 µL of plasma was mixed with 290 µL of reaction mixture, and the concentration was measured in the microplate reader at 340 nm following a 30-min incubation at room temperature. Plasma pH was measured by an electrolyte analyzer based on ion-selective electrode technology (Convergys ISE comfort Electrolyte Analyzer, Convergent Technologies, Cölbe, Germany). Plasma osmolality was measured using a cryoscopic osmometer (Advanced Model 3320 Micro-Osmometer4, Advanced Instruments Inc., Norwood, USA).

### Gill NKA-ATPase activity

Gill NKA-ATPase activity was measured using the kinetic enzyme assay developed by McCormick (1993). In brief, 2–3 gill filaments were homogenized in 200 µL SEI-buffer with 0.1% deoxycholic acid using a hand-held battery-operated pellet mixer (VWR International, Radnor, USA) for 1 min on ice. The homogenate was centrifuged (13.300 × g at 4 °C for 8 min), and the supernatant was extracted and used for further analysis. The absorbance readings of NADH were performed in a microplate reader (SpectraMax 190, Molecular Devices Corp., Menlo Park, USA) at 340 nm for 10 min at 25 °C. Specific NKA-activity is expressed as nmol ADP/µg protein/h. Protein concentrations of the homogenates were measured using the bicinchoninic acid (BCA) protein assay kit according to the manufacturer’s instructions (Pierce, Rockford, USA).

### Ussing chamber methodology

The intestinal function was assessed using the Ussing chamber technique described in detail by Sundell et al. (2003) using modifications further described by Sundell and Sundh (2012). The proximal and distal intestines were stripped of the serosa and muscular layer under a stereo microscope on a chilled dissection plate and subsequently mounted in chambers to which 4 mL Ringer’s solution was added to each half-chamber. The transepithelial resistance (TER), trans-epithelial potential (TEP), and short circuit current (SCC) were measured across the epithelia while using identical Ringer’s solutions on the mucosal and serosal side with a 0.75 cm^2^ exposure area. The tissue was aerated through a gas-lift, and a cooling mantel with circulating water at 10 °C was applied to maintain the environmental holding temperature. Measurements were performed with 5-min intervals to avoid epithelial capacitance building. The intestines were allowed to acclimate for 60 min to reach a steady state. After 60 min, the Ringer’s solution was exchanged in both chambers; the serosal side received new fresh Ringer’s solution, and the mucosal side received Ringer’s solution containing the marker molecule ^14^C-mannitol (142 Bq/µL; PerkinElmer, Waltham, USA) and ^3^H-methionine (164 Bq/µL; PerkinElmer, Waltham, USA) in the presence of 0.5 mmol/L unlabeled methionine. ^14^C-mannitol was used to assess epithelial permeability and ^3^H-methionine to assess the nutrient transport. The rate of radioactivity accumulation over 90 min was calculated from the radio in 100 µL of Ringer’s solution withdrawn from the mucosal and serosal chamber at T = 0 and subsequent 100 µL samples that were taken from the serosal chamber at *T* = 20, 25, 30, 60, 80, 85, and 90 min. Each sample was exchanged for 100 µL fresh Ringer’s solution to avoid differences across the epithelium in hydrostatic pressure. Scintillation fluid (5 mL, Ultima Gold, PerkinElmer, Waltham, USA) was added to each 100-µL sample, and radioactivity was determined using a β-counter (Wallac 1409 Liquid Scintillation Counter, Turku, Finland). The apparent permeability coefficient (*P*_app_) for mannitol, the mucosal to serosal methionine transport (methionine transport) across the epithelium was calculated using the following equations:

$${P}_{\text{app}}=((\text{dQ}/\text{dt}) \times (1/{\text{AC}}_{0}) )$$

where dQ/dt is the serosal accumulation of ^14^C-mannitol over time (mol/min), *C*_0_ is the initial concentration on the mucosal side (mol/mL), and *A* is the exposed tissue area (*A* = 0.75 cm^2^). If a rapid reduction in TER and a corresponding rapid increase of *P*_app_ was observed, this was interpreted as tissue damage, and the sample was excluded from the analyses.

$$Methionine\, transport = (dQ/dt) / A$$

where dQ/dt is the accumulation of methionine over time (mol/min) and *A* the intestinal tissue area (*A* = 0.75 cm^2^).

### Statistical analysis

The software used to perform the statistical analysis was GraphPad Prism (GraphPad Prism version 9.3.1 for Windows, GraphPad Software, Boston, USA). All data was assessed for parametric testing using QQ plots, the Brown–Forsythe test for equal variances, and the Shapiro–Wilk test for normal distribution. One-way ANOVA was used to analyze the data for plasma cortisol, NKA activity, and blood variables. To analyze the data from the Ussing chambers (TER, TEP, SCC, P_app_, and methionine transport), a two-way ANOVA, using treatment and intestinal regions as fixed factors, with a post hoc Šídák’s multiple comparison test was used.

## Results

**Fig. 1:**
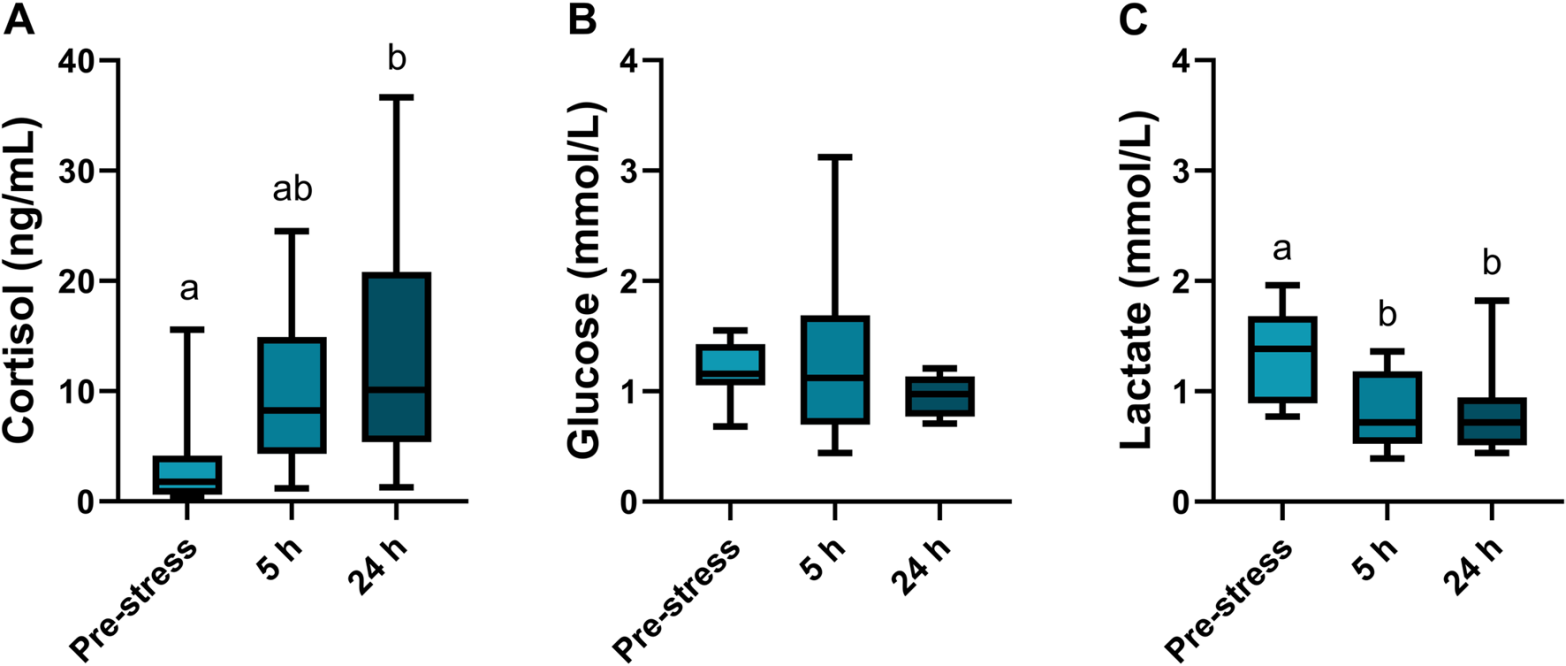
Plasma concentration of **A** cortisol (ng/mL), **B** glucose (mmol/L), and **C** lactate (mmol/L) in Atlantic wolffish pre-stress, 5 h and 24 h following acute handling stress (*n* = 10). Data are presented as a box plot containing the median value delineated by the interquartile range (1st to 3rd quantile) and an accompanying whisker that represents the minimum and the maximum values. Different letters denote significant differences

**Fig. 2:**
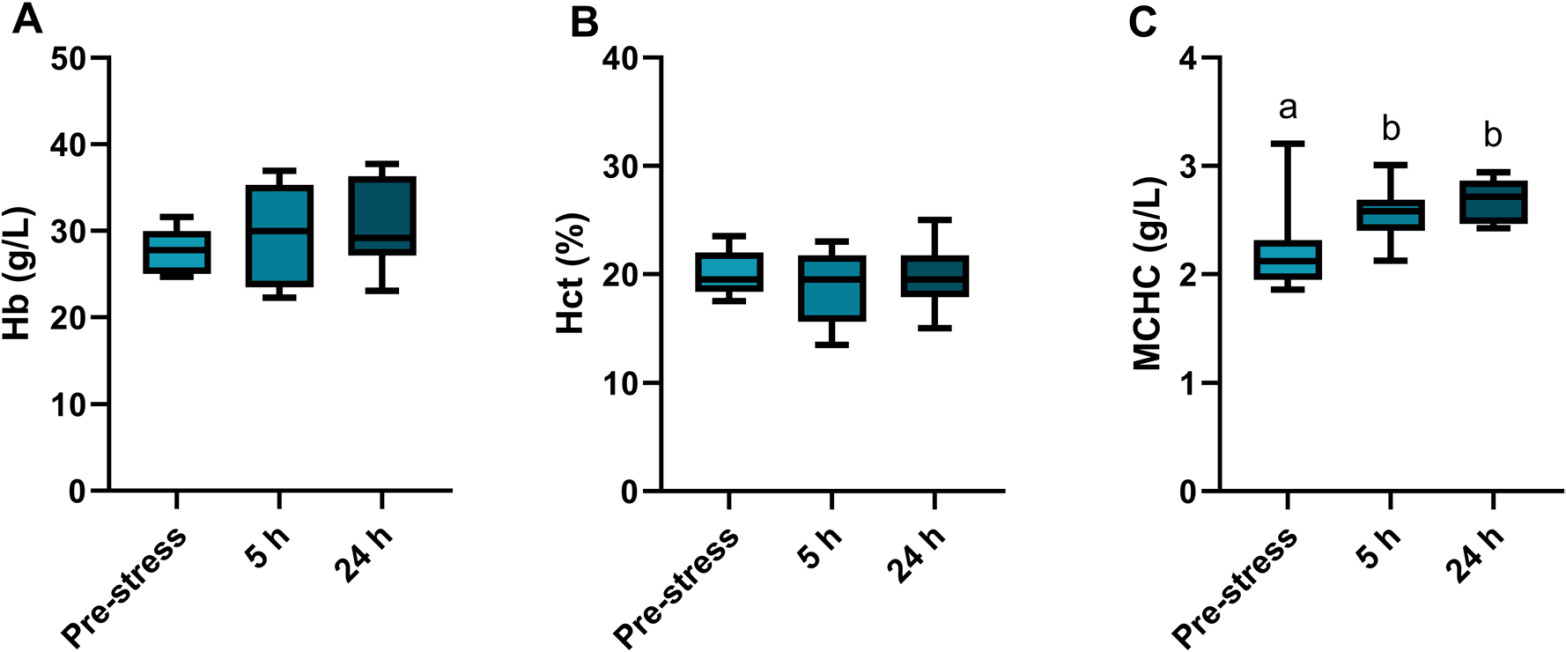
Hematological indices **A** hemoglobin concentration (Hb, g/L), **B** hematocrit (Hct, % red blood cells), and **C** mean corpuscular hemoglobin concentration (MCHC, g/L) in Atlantic wolffish pre-stress, 5 h and 24 h following acute handling stress (*n* = 10). Data are presented as a box plot containing the median value delineated by the interquartile range (1st to 3rd quantile) and an accompanying whisker that represents the minimum and the maximum values. Different letters denote significant differences

The acute stress exposure resulted in a primary stress response measured as a gradual increase in plasma cortisol concentrations reaching the highest concentration, 13.55 ± 3.56 ng/mL (average ± standard error), after 24 h (one-way ANOVA *p* = 0.03; Fig. 1A). The acute stress did not affect the plasma concentrations of glucose at the time points measured (one-way ANOVA *p* = 0.31; Fig. 1B). There was a significant reduction in plasma lactate concentration after 5 h, which remained low after 24 h (one-way ANOVA *p* = 0.01; Fig. 1C). The plasma pH, plasma osmolality, and branchial NKA activity were not affected by the single acute stress exposure (one-way ANOVA; *p* = 0.78, *p* = 0.85, and *p* = 0.15, respectively, Table 1). The assessment of the blood parameters showed no changes in Hb (one-way ANOVA *p* = 0.39; Fig. 2A) or Hct (one-way ANOVA *p* = 0.67; Fig. 2B). However, the calculated MCHC showed that there was a significant increase 5 h after stress exposure, which was also maintained after 24 h (one-way ANOVA: *p* < 0.01; Fig. 2C).

**Table 1:** Gill NKA-activity, plasma pH and plasma osmolality in Atlantic wolffish pre-stress, 5 h and 24 h following acute handling stress (*n* = 10). Data are presented as mean ± standard error

Treatment	NKA activity nmol ADP/mg protein/h	Plasma pH	Plasma osmolality (mOsmol/kg)
Pre-stress	0.99 ± 0.07	7.38 ± 0.02	311.9 ± 1.3
5 h	0.74 ± 0.05	7.37 ± 0.02	311.1 ± 2.5
24 h	1.17 ± 0.26	7.36 ± 0.02	312.9 ± 2.7

**Fig. 3:**
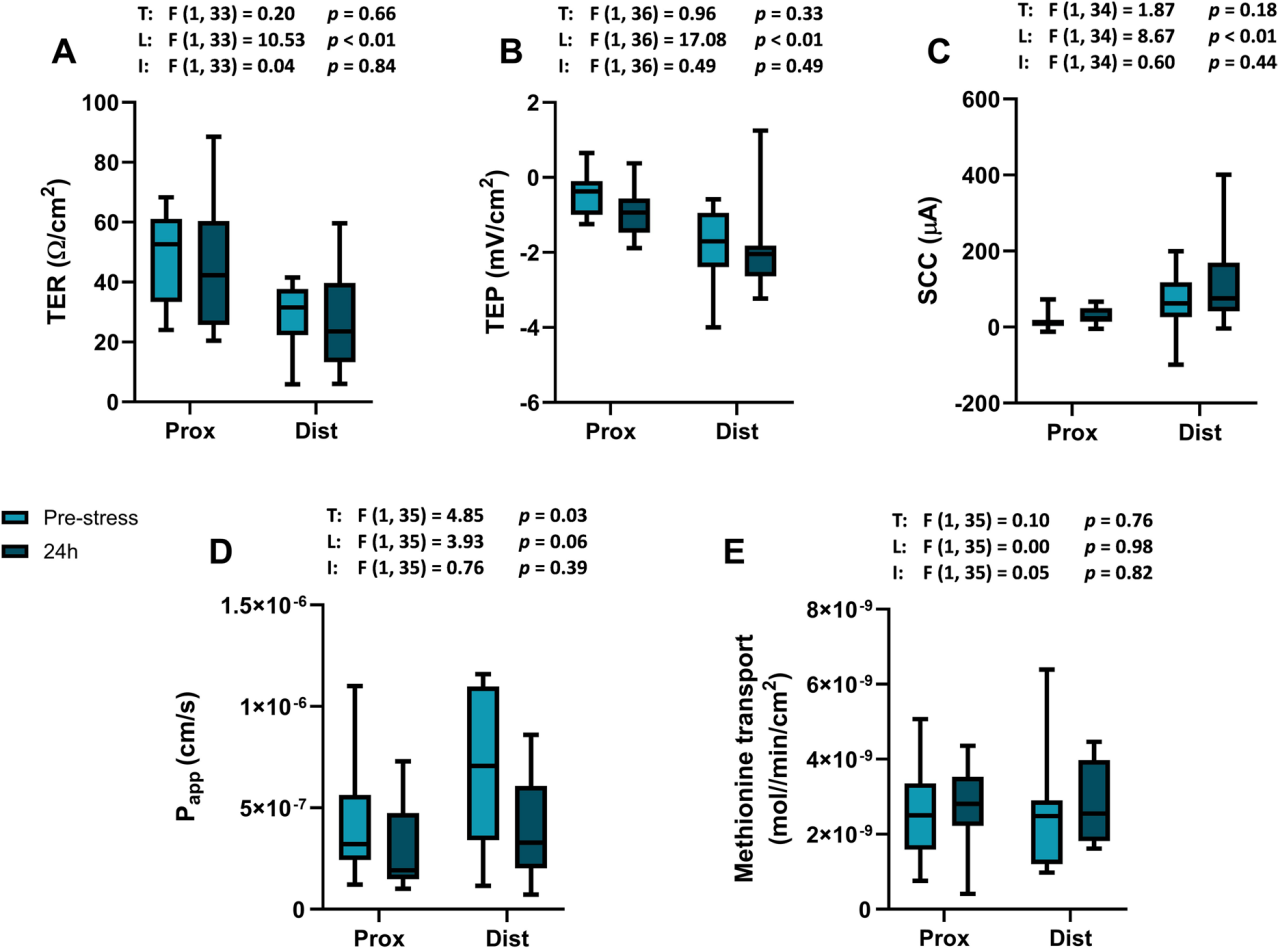
Ussing chamber measurements of the intestinal barrier and transport function: **A** transepithelial resistance (TER), **B** transepithelial potential (TEP), **C** short circuit current (SCC), **D** mannitol diffusion rate (Papp), and **E** methionine transport in Atlantic wolffish pre-stress and 24 h respectively following acute handling stress (*n* = 10). Data are presented as a box plot containing the median value delineated by the interquartile range (1st to 3rd quantile) and an accompanying whisker that represents the minimum and the maximum values. T, main effect of time (pre-stress vs. 24 h); L, main effect of location (proximal vs. distal). I, interaction effect. Prox, proximal; Dist, distal

The Ussing chamber measurements revealed that acute stress exposure had no effect on the intestinal integrity measured as TER (two-way ANOVA *p* = 0.66; Fig. 3A). There was, however, a significant difference between the intestinal regions where the proximal intestine had higher TER in comparison to the distal intestine (two-way ANOVA *p* < 0.01; Fig. 3A). Assessment of the ion-transport activity measured as TEP (two-way ANOVA *p* = 0.33; Fig. 3B), and SCC (two-way ANOVA *p* = 0.18; Fig. 3C) showed that this function was not affected by the stress treatment. As observed for the TER, there was a significantly lower TEP (two-way ANOVA *p* < 0.01; Fig. 3B) and a significantly higher SCC (two-way ANOVA *p* < 0.01; Fig. 3C) in both proximal and distal intestine sections. Mannitol permeability, measured as *P*_app_, was significantly affected by stress (two-way ANOVA *p* = 0.03; Fig. 3D), and the post hoc comparison (Šídák's multiple comparisons test; *p* = 0.06) revealed a tendency toward decreased permeability in the distal intestine 24 h after stress. Methionine transport was not affected by the exposure to acute stress (two-way ANOVA *p* = 0.76; Fig. 3E).

## Discussion

The current work shows for the first time that mimicking potentially recurrent stressors encountered in aquaculture, such as acute air exposure and chasing, elicited a stress response in Atlantic wolffish within the 24 h following the stress event, as reflected by subtle, but significant, physiological changes.

Atlantic wolffish displayed a slow and low increase in plasma cortisol levels following an acute stress exposure, with cortisol levels gradually increasing from 3.29 ± 1.43 to 13.55 ± 3.56 ng/mL (average ± standard error) during the first 24 h post-stress, as compared to those reported for several aquaculture species where cortisol concentrations below 5 ng/mL are considered non-stressed (or basal) level (Barton 2002; Barton and Iwama 1991). This study, together with other studies on Atlantic wolffish and its close relative, the spotted wolffish, confirm that non-stressed wolffish also have basal cortisol concentrations within this range, with levels of 3–6 ng/mL reported (Lays et al. 2009; Le François et al. 2013).

There was a slight increase in plasma cortisol 5 h post-stress that continued, to reach the highest concentrations after 24 h. These results are in line with what was observed in the spotted wolffish, where the cortisol peak was apparent at 3–6 h for juveniles and at 13 h in adult fish (Lays et al. 2009; Le François et al. 2013). In addition, both wolffish species continue to have elevated cortisol levels 24 h post-stress and up to 37 h in spotted wolffish (Le François et al. 2013). Hence, with the knowledge of spotted wolffish together with the results from the present study, it can be speculated that the cortisol peak for Atlantic wolffish may have occurred between 5 and 24 h post-stress. Further studies, with higher temporal resolution, are needed to pinpoint the cortisol peak and understand the duration of its release following acute stress in this wolffish species.

The timing and intensity of the cortisol response in fish can vary greatly depending on the species, its environment, and lifestyle. Cold-water Arctic and Antarctic fish species are known to have low cortisol peaks (below 65 ng/mL) compared to more temperate fish like salmonids (Whiteley et al. 2006; Whiteley and Egginton 1999). This could be a result of low metabolic rates, the stability of their environments, evolutionary adaptations, alternative mechanisms for coping with stress, a high tolerance to stress, or a combination of those. Lower cortisol levels are also generally observed in benthic, sedentary fish, compared to pelagic, more active species (Silkin and Silkina 2005). For example, transportation but not air exposure caused a minor increase of cortisol in the pallid sturgeon (*Scaphirhynchus albus*, around 5 ng/mL) (Barton et al. 2000). Furthermore, confinement stress in pallid sturgeon and hybrids between pallid × shovelnose sturgeon (*S. albus* × *S. platorynchus*), resulted in only minor plasma cortisol increases to about 15 ng/mL at 1 h post-stress, which returned to pre-stress levels within 24 h (Barton et al. 2000). In contrast, cortisol levels in salmonids following acute stress exposure can typically reach several hundred ng/mL typically within 1–6 h (Barton 2000; Barton and Iwama 1991).

The current study suggests that Atlantic wolffish, like spotted wolffish, are slow and low cortisol responders. This can probably be attributed to their calm behavior and sedentary benthic lifestyle as seen in other low cortisol responders (Barton 2002; Foss et al. 2004; Rountree 2002). Prolonged or repeated exposure to stressors can result in an overstimulation of the HPI axis in fish, reducing or disabling further cortisol release, potentially refraining from new acute stress responses (Sundh et al. 2019; Wendelaar Bonga 1997). However, this explanation for a low cortisol response in this study is unlikely as the animals have been kept in the same system under standardized husbandry routines and stable environmental conditions with good water quality for several years (Hedén 2023).

Despite the low cortisol release from wolffish following acute stress, these fish appear to have the capacity to elevate plasma cortisol above the maximum levels observed in this study (37 ng/mL). Similarly to other low cortisol responding species, such as the white sturgeon (*Acipenser transmontanus*), ACTH injection resulted in high plasma levels cortisol levels in spotted wolffish (above 150 ng/mL) (Belanger et al. 2001; Lays et al. 2009). The relatively weak cortisol response seen in wolffish species could therefore also reflect a low HPI axis reactivity toward stressors (Sumpter et al. 1986). The production of cortisol would trigger a negative feedback loop which reduces the secretion of CRH and ACTH, which in turn slows down the production of cortisol (Wendelaar Bonga 1997). It would therefore be of high interest to assess the dynamics of the releasing hormones of the HPI axis in Atlantic wolffish, along with the abundance, affinity, and turnover rate of the cortisol receptors. This would allow a more comprehensive view of the primary stress response and its regulation in this species.

Even though plasma cortisol is considered a reliable acute stress indicator (Sadoul and Geffroy 2019), it is hard to draw conclusions on the stress response with only one indicator. To understand how stressors affect fish, it is therefore important to also use and evaluate multiple indicators for secondary stress responses. Hematological indices are commonly used in welfare studies on stress and respiratory physiology (Fazio 2019). In the current study, the blood analyses of Hb and Hct alone did not show any signs of stress-related change. There was a tendency toward increased Hb concentrations after stress exposure while the Hct remained relatively stable (on average 20%). The slight increase in Hb together with stable Hct resulted in an increased MCHC after 5 h and 24 h. MCHC normally decreases after stress because of increased concentration of erythrocytes (higher Hct) released from the spleen or due to red blood cell swelling lowering the Hb (Wendelaar Bonga 1997). In this case, the stable Hct combined with increased Hb suggests a different mechanism at play, potentially indicating an increase in wolffish Hb synthesis rather than an increased number of red blood cells. The Hb concentration and Hct in wolffish measured in this study are relatively low in comparison to commonly farmed fish species such as African catfish (*Clarias gariepinus*), common carp (*Cyprinus carpio*), European seabass (*Dicentrarchus* *labrax*), Gilhead seabream (*Sparus aurata*), Nile tilapia (*Oreochromis niloticus*), and rainbow trout (*Onchorhynchus mykiss*) (Fazio 2019). Hematological measurements are influenced by numerous factors such as sex, species, environmental cues, stress, and disease (Ahmed et al. 2020; Fazio 2019), indicating that further evaluation is needed to fully understand how the wolffish’s hematological indices are affected by stress. Such knowledge can be used to determine baselines for these parameters for the different life stages of the species in order to establish tools for the aquaculture industry for monitoring fish welfare.

Glucose and lactate are common indicators of secondary stress responses (Barton 2002; Wendelaar Bonga 1997). Generally, there is first an increase in glucose and lactate concentrations after a stress exposure, which is then reduced over time (Barton 2002; Fanouraki et al. 2011; Jiang et al. 2017; Pottinger 1998). In this study, Atlantic wolffish did not show any changes in plasma glucose levels while there was a significant reduction in plasma lactate 5 and 24 h after stress exposure. The lack of response in glucose concentration does not necessarily mean that there was no increase at an earlier point. Comparative studies have shown varying responses for different species regarding the timing of the glucose and lactate responses (Fanouraki et al. 2011). Similarly to the hematological indexes and the plasma cortisol levels, plasma glucose and lactate levels of Atlantic wolffish were relatively low compared to other commonly farmed species, including salmonids (Barton 2000; Remen et al. 2012). The glucose response could be divided into two general patterns where fish can have a fast increase (within 1 h) after the stress exposure that rapidly falls to pre-stress levels or that glucose concentrations are more or less elevated with slow decrease back to pre-stress levels over 24 h. The lactate response could also be divided into two general patterns with an initial increase that is maintained at a higher concentration over 24 h or with a fast reduction within 5 h (Fanouraki et al. 2011). The current results suggest that the wolffish may experience a fast initial increase in glucose within the first hours following stress, with levels returning to control levels within 5 h. Lactate concentrations, on the other hand, were reduced within 5 h and remained low after 24 h.

Cortisol, along with other hormones, is known to induce osmoregulatory responses in fish to maintain homeostasis regarding salt- and water balance which may lead to changes in gill NKA-activity as one outcome (Takahashi and Sakamoto 2013; Takei and McCormick 2012). It has been suggested that gill NKA activity increases after a stressor to counteract a possible disturbance of the hydromineral homeostasis as a result of stress-induced decreases in barrier functions (Barton and Iwama 1991; Benktander et al. 2021). Cortisol-induced elevations in NKA protein levels are considered to take some time since they are classically regulated through gene expression, cell proliferation, and differentiation (Takei and McCormick 2012). Although the exact mechanisms remain unclear, there is also in vivo and in vitro evidence of more rapid, non-genomic actions of these hormones in branchial NKA activity (Chang et al. 2023). In freshwater species, such as African catfish and Mozambique tilapia (*Oreochromis mossambicus*), the gill NKA-activity was found to rapidly increase (within 20–30 min) in response to cortisol administration through perfusion or injection (Babitha and Peter 2010; Sunny and Oommen 2001). In the current study, the cortisol concentration was highest after 24 h, at which time some individuals displayed a slightly increased gill NKA activity as well. Despite this small inter-individual variability, there was no difference between the different time points in this study, suggesting that there was no evidence for any rapid non-genomic actions of cortisol on gill NKA activity. Nevertheless, it cannot be excluded that there could have been an elevated NKA activity beyond 24 h if NKA protein synthesis had been induced. For instance, a chronic (50-day) exposure to high temperature led to a significant increase in branchial NKA-activity in Atlantic wolffish, while an acute exposure (24 h) did not result in any changes (Hinchcliffe et al. 2025a).

The intestinal barrier functions are known to be affected by various forms of stress, both in mammals (Söderholm and Perdue 2001) and in fish (Sundh and Sundell 2015). In salmonids for instance, it has been reported that different forms of stressors such as handling, temperature elevation, and crowding negatively affect the intestinal barrier function resulting in a more leaky intestine with decreased TER and increased P_app_ (Olsen et al. 2005, 2008; Rosengren et al. 2018; Sundh et al. 2018, 2010, 2009). These changes can lead to compromised nutrient absorption, increased risk of infections due to microbial translocation, and disturbances to the immune system, all of which can have broad physiological consequences and impact overall health (Warwas et al. 2024). Increased leakiness of the intestine has further been connected to elevated cortisol concentrations (Sundell and Sundh 2012). However, in the current study, acute stress did not affect the intestinal barrier or transport functions in Atlantic wolffish. Our results herein are also in agreement with a previous study on Atlantic wolffish, where acute exposure to an elevated temperature did not affect neither cortisol levels nor intestinal barrier or transport functions (Hinchcliffe et al. 2025a). Together with the blood and plasma physiological responses, these results indicate that Atlantic wolffish do not appear to be highly sensitive to acute stressors, potentially encounterable in aquaculture setups. Future study should investigate the precise timing of the cortisol peak and the full recovery time from acute stressors to better understand the stress-coping abilities Atlantic wolffish in aquaculture settings.

## Summary

This study showed that Atlantic wolffish had a slow increase in plasma cortisol concentration in response to handling, with the highest levels reached after 24 h. There was no effect on plasma glucose levels, while plasma lactate levels decreased after 5 h and remained low until 24 h. Hematological indices, plasma pH and osmolality, and gill NKA activity were not affected by acute stress. Furthermore, the intestinal barrier and transport functions remained unaffected. Our conclusion is that Atlantic wolffish exhibits a relatively slow and low cortisol response, with few secondary stress responses within 24 h. This may be related to their sedentary lifestyle and known docile behavior, which, along with other traits, are important considerations when evaluating wolffish as a candidate for development and diversification of marine, cold-water aquaculture.

## Data Availability

No datasets were generated or analysed during the current study.
